# Bridging Language Barriers in COVID-19 Research: Descriptive Study of AccesoCovid.com’s Reach and User Engagement

**DOI:** 10.2196/53978

**Published:** 2024-09-09

**Authors:** Lucía Abascal Miguel, Maeve Forster, Sarah Gallalee, Mariam Carson, Jane K Fieldhouse, Alexandra Keir, Sigal Maya, Sabahat Rahman, Michael J A Reid, Hariclea Vasilopoulos, Dania Nimbe Lima Sanchez

**Affiliations:** 1 Institute for Global Health Sciences University of California, San Francisco San Francisco, CA United States; 2 Philip R. Lee Institute for Health Policy Studies University of California, San Francisco San Francisco, CA United States; 3 Department of Biomedical Informatics School of Medicine Universidad Nacional Autónoma de México Mexico City Mexico

**Keywords:** COVID-19 research dissemination, multilingual scientific platform, language barriers in science, Spanish scientific communication, equitable access to research, global health equity

## Abstract

**Background:**

The COVID-19 pandemic underscored the challenge of swiftly disseminating research findings to a global audience. Language barriers further exacerbated disparities in access to timely scientific information, particularly for non-English speaking communities. The majority of COVID-19 research was published in English, limiting accessibility for Spanish-speaking populations.

**Objective:**

This paper aims to assess the reach and effectiveness of AccesoCovid.com, a platform designed to disseminate up-to-date COVID-19 research in both English and Spanish, addressing the language gap in scientific communication.

**Methods:**

AccesoCovid.com was developed through a partnership between the University of California, San Francisco (UCSF) and Universidad Nacional Autónoma de México (UNAM). The website’s performance and user engagement were evaluated using Google Analytics over a span of 2 years. Key metrics included user language preference, geographical distribution, and site traffic. The website summarized and translated 1032 articles on various COVID-19 topics, such as “Pharmaceutical Interventions and Vaccines.”

**Results:**

From February 2021 to February 2023, the platform attracted 57,000 users. Of the 43,000 unique new visitors, 84.2% (n=36,219) hailed from Spanish-speaking regions. The majority accessed the site organically through search engines, with 88.4% (n=38,000) of users arriving this way, while 5000 (11.6%) users accessed the site directly. Most users (n=30,894, 72.1%) preferred the Spanish version of the site. The website’s most accessed category was “Pharmaceutical Interventions and Vaccines,” followed by “Clinical Presentation and Management” and “Mental Health.” Regarding language distribution, 72.1% (n=30,894) of users primarily used Spanish; 21.4% (n=9215) used English; and 6.7% (n=2891) spoke other languages, including Portuguese, Chinese, and German. Geographically, the website attracted visitors from 179 countries, with the highest visitor counts from Mexico (n=12,342, 28.7%), Spain (n=6405, 14.9%), the United States (n=4416, 10.3%), and Peru (n=3821, 8.9%).

**Conclusions:**

AccesoCovid.com successfully bridged a critical language gap in the dissemination of COVID-19 research. Its success underscores the pressing need for multilingual scientific resources. The platform demonstrated significant user engagement and reach, particularly in Spanish-speaking countries. This highlights the potential for similar platforms to ensure equitable access to scientific knowledge across diverse linguistic communities. Future efforts should focus on expanding to other languages and conducting formal evaluations to enhance user satisfaction and impact.

## Introduction

The rapidly evolving COVID-19 pandemic made it challenging yet crucial for policy makers and health professionals to stay informed. Comprehensive understanding of the latest COVID-19 research was essential for developing health policies, conducting timely research, and implementing clinical practices [1]. Yet the large volume and high frequency of peer-reviewed publications during the height of the pandemic made it difficult to fully synthesize emerging research [2].

Language barriers may have prevented both the public and professionals from accessing informative publications [3]. Despite the global demand for health information, most literature is published in English, a language spoken natively by around 380 million people (5% of the global population), with up to 20% of nonnative speakers [1,4]. In contrast, Spanish is spoken natively by 500 million people (6% of the global population), with 74 million speaking it as a second language [5]. In the first year of the pandemic, 98% of COVID-19 articles indexed in PubMed and the National Library of Medicine were in English, while only 0.66% were in Spanish [6]. Effective access to science is crucial, as it influences clinical decision-making and enhances understanding of public health interventions and treatment availability [7].

The importance of translating scientific research to bridge language barriers is well recognized, with platforms such as TranslateScience.org and numerous researchers advocating for expanded accessibility. Despite these efforts, there remains a significant gap in the global dissemination of scientific knowledge [8]. To address the lack of COVID-19 scientific literature in Spanish, a binational partnership between the University of California, San Francisco (UCSF) and Universidad Nacional Autónoma de México (UNAM) created the AccesoCovid project in September 2020. This platform is a searchable, open-access website that hosts COVID-19 research summaries available in both Spanish and English. This paper assesses the reach and effectiveness of the website 3 years after its launch.

## Methods

### AccesoCovid Website Overview

The website was developed on the Webflow platform with support from a web designer from Mexico. Two URLs, “AccesoCovid.com” and “AccessCovid.com,” were used to direct users to the same web page.

**Figure 1:**
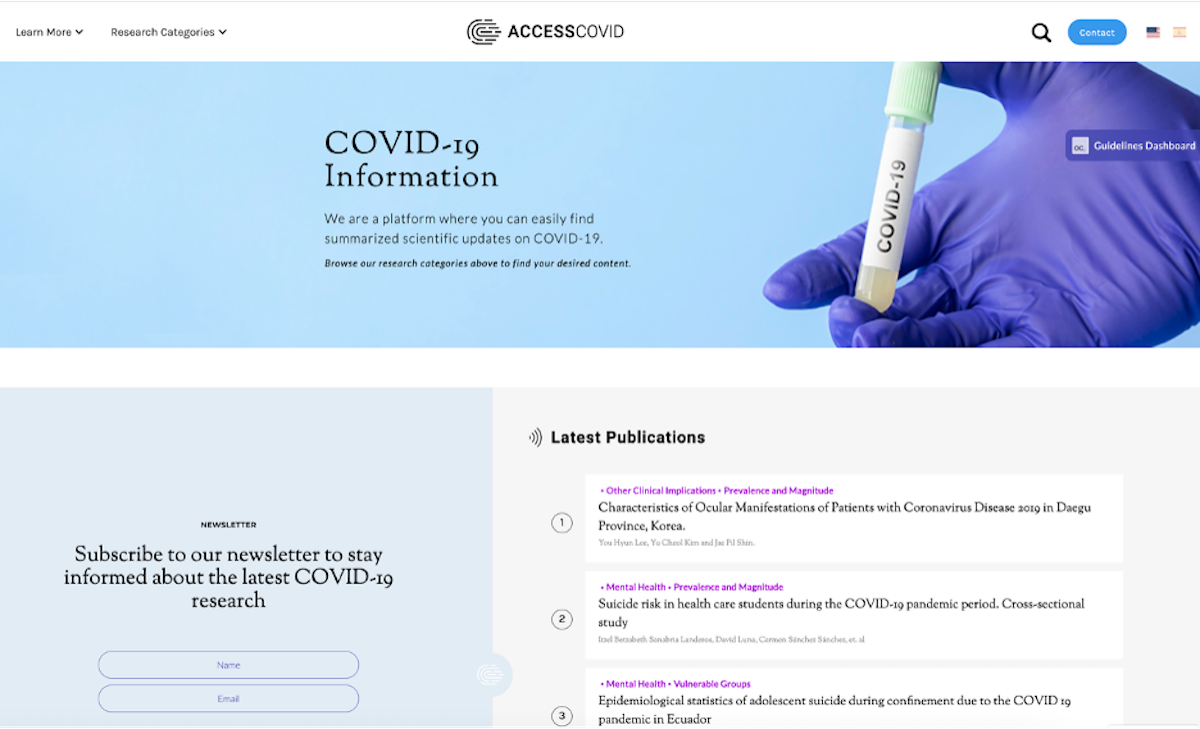
Screenshot of the landing page of AccesoCovid.com.

The landing page of the website allows users to choose their preferred language, English or Spanish. Users are then presented with the latest and most popular summaries on the home page (Figure 1). A search engine feature enables users to further explore the summaries using keywords, dates, or categories. This website continues to serve as a gateway for the general public to access lay summaries along with publication metadata and URLs.

The website was advertised to UCSF personnel through internal UCSF resources as well as through UNAM’s networks. It was also advertised to the Global Health community through organizational social media and included as a resource for participants of the Strengthening Interprofessional Education for HIV (STRIPE HIV) training program. AccesoCovid did not engage in paid advertisements.

### AccesoCovid Teams

Volunteer students from the UCSF; University of California, Los Angeles; and UNAM supported this project as editors, who selected and edited articles, or summarizers, who read and summarized them. A total of 44 volunteers were part of the UCSF team and 36 were from the UNAM team. Volunteers fluctuated based on availability.

### AccesoCovid Content Development

Summaries were initially written in English or Spanish, translated, and then added to the website through the following steps:

1. Twice a week, editors conducted searches on the PubMed or on individual journal websites and selected pertinent and recently published scientific articles in either English or Spanish on COVID-19–related topics (Multimedia Appendix 1);
2. Selected articles were reviewed by clinicians and public health researchers who weighed in on the most relevant articles;
3. Summarizers read the selected articles and drafted 4- to 5-sentence summaries encapsulating the main points of each selected article;
4. At least 2 editors reviewed the drafted summaries for clarity and accuracy;
5. Editors added the reviewed summaries to a shared Google Sheet, along with the article’s metadata (eg, title, authors, and date);
6. Either native Spanish or English speakers translated the summaries and added the translations to the shared Google Sheet;
7. Once a week, a member of the UNAM team uploaded the newly added content from the shared Google Sheet to the website. Each article was categorized and indexed on the website.

### AccesoCovid Evaluation

Google Analytics was used to analyze the website data to assess the project’s effectiveness. Data from February 9, 2021, to February 9, 2023, were included. Site-specific metrics including the number of total and unique users, user language, geographical distribution of users, and category of content accessed were evaluated.

### Ethical Consideration

This study is exempt from institutional review board review by UCSF since it does not involve human subjects (study 24-41940; reference 411495).

## Results

The website officially launched on February 9, 2021. Between February 9, 2021, and February 9, 2023, the website attracted a total of 57,000 users, of which 43,000 (75.4%) were unique new visitors.

### Content Produced

A total of 1032 articles were summarized, edited, and translated between February 9, 2021, and February 9, 2023. The category with the greatest number of summaries was “Pharmaceutical Interventions and Vaccines,” closely followed by “Clinical Presentation and Management” and “Mental Health.” The list of categories can be found on Multimedia Appendix 1.

### User Acquisition

Most users (38,000/43,000, 88.4%) arrived at the web page organically, reaching the site through search engine results—a listing on Google Search that appears due to its relevance to users’ search terms. An additional 5000 (11.6%) users accessed the website directly using the URLs.

### Language Distribution

Browser language was used as a proxy to analyze the language distribution among the user base. Out of the 43,000 users, 30,894 (72.1%) individuals predominantly used Spanish, while 9215 (21.4%) used English. Additionally, 2891 (6.7%) users spoke a primary language other than Spanish or English, with Portuguese, Chinese, and German being the most commonly represented among a total of 48 different languages.

### Geographical Distribution

**Figure 2:**
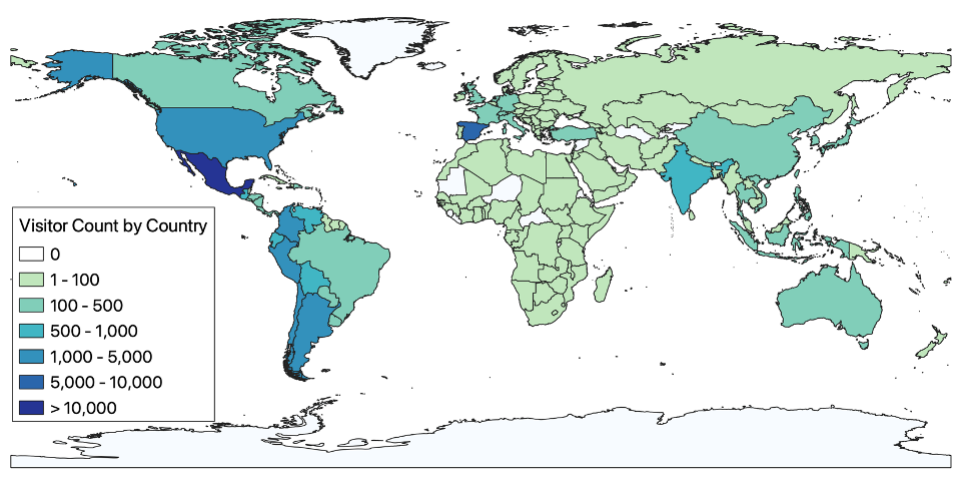
Geographic distribution of visitors to AccesoCovid.com from February 2021 to February 2023.

The website attracted visitors from 179 distinct countries (Figure 2). The countries with the highest visitor counts were Mexico, accounting for 12,342 (28.7%) visitors, followed by Spain with 6406 (14.9%) visitors, the United States with 4416 (10.3%) visitors, and Peru with 3821 (8.9%) visitors. A total of 84.2% (n=36,219) of visitors were in Spanish-speaking countries in Latin America.

## Discussion

AccesoCovid.com quickly distributed up-to-date research in English and Spanish during the COVID-19 pandemic, bridging the language gap in COVID-19 research. We had 57,000 users during the first 2 years of the website and 84.2% (36,219/43,000) of our visitors came from Spanish-speaking regions, emphasizing the significance of our efforts. We summarized research into simplified language for public accessibility and translated it into Spanish, aiming to promote scientific information for evidence-based decisions. It is an effective example of a collaboration between academic partners, UNAM and UCSF, to rapidly meet a global and evolving need.

The success of our website, measured as the overall and organic traffic, showcases a substantial need for accessible, multilingual scientific information. In a landscape where most health-related literature is published in English, our findings are expected and consistent with existing work that highlights disparities in scientific communication [1]. English dominates scientific publishing, especially in the natural sciences, where over 90% of papers are in English [9]. This marginalizes other major languages, creating barriers for nonnative speakers to consume and produce scientific literature, thus impacting equitable access to scientific information and leading to disparities in publishing outcomes [10]. Although the primary focus of AccesoCovid.com has been to support Spanish speakers’ access to COVID-19 research, this approach can be expanded to other languages or subject areas. As such, the website has universal application to support countries and populations grappling with language barriers in accessing scientific literature.

Some limitations of our study include the rapid development of the repository, which precluded user experience testing or assessing the website’s effectiveness with our target audience. Additionally, while the descriptive statistics obtained via Google Analytics provide preliminary insights, they do not constitute a formal evaluation. Key data such as user access by article category and user demographics were not available. Future efforts should use robust evaluation methods, including user surveys and interviews, to effectively assess user satisfaction and experience, gain deeper insights into how the website is used, and gather feedback for improvements. Nevertheless, the data on user visits and engagement strongly suggest that the site successfully addresses a previously unmet need.

The evident need for accessible scientific literature calls for a global effort to ensure that non-English speakers benefit from scientific knowledge. Systemic changes should be encouraged to make science accessible in more languages as well as in lay terms. Changes may include ways to allow research to be published in the author’s first language, mechanisms for research to be systematically translated, or forums that provide easier access by the public. Enhancing access to scientific literature can foster better-informed public health decisions, inclusive research collaborations, and a more equitable distribution of knowledge.

## Appendix

Multimedia Appendix 1 Categories of articles, journals searched, and PubMed query parameters used by editors to select COVID-19 research summaries from September 2020 to February 2023.

## Abbreviations

STRIPE HIV: Strengthening Interprofessional Education for HIV
UCSF: University of California, San Francisco
UNAM: Universidad Nacional Autónoma de México

## Data Availability

Google Analytics data are available upon request to the corresponding author.

## References

[1] Pakenham-Walsh N, Healthcare Information For All working group on multilingualism (2018). Improving the availability of health research in languages other than English. Lancet Glob Health.

[2] Else H (2020). How a torrent of COVID science changed research publishing - in seven charts. Nature.

[3] Deng B (2015). English is the language of science. Slate.

[4] Hommes F, Monzó HB, Ferrand RA, Harris M, Hirsch LA, Besson EK, Manton J, Togun T, Roy RB (2021). The words we choose matter: recognising the importance of language in decolonising global health. Lancet Glob Health.

[5] Albares J (2023). Spanish is the second mother tongue worldwide and the second language in terms of international communication. Ministry for Foreign Affairs, European Union and Cooperation.

[6] Kang M, Gurbani SS, Kempker JA (2020). The published scientific literature on COVID-19: an analysis of PubMed abstracts. J Med Syst.

[7] Pelicioni PHS, Michell A, Santos PCRD, Schulz JS (2023). Facilitating access to current, evidence-based health information for non-English speakers. Healthcare (Basel).

[8] Editorial (2023). Scientific publishing has a language problem. Nat Hum Behav.

[9] Berdejo-Espinola V, Amano T (2023). AI tools can improve equity in science. Science.

[10] Gomez CJ, Herman AC, Parigi P (2022). Leading countries in global science increasingly receive more citations than other countries doing similar research. Nat Hum Behav.

