# Supplementary material for: Bridging Language Barriers in COVID-19 Research: Descriptive Study of AccesoCovid.com’s Reach and User Engagement
Source: JMIR Form Res. 2024 Sep 9;8:e53978. doi: 10.2196/53978 (PMC11420591; doi:10.2196/53978)
Supplement: Multimedia Appendix 1 [file formative_v8i1e53978_app1.docx]

**Table 1.** Categories of articles, journals searched, and PubMed query parameters used by editors to select COVID-19 research summaries from September 2020 to February 2023.

| Categories | Bioethics Bioengineering Clinical Presentation and Management Diagnostic and Serological Tests Mental Health Models and Forecasting Non-Clinical Trends Non-Pharmaceutical/Public Health Interventions Other Clinical Implications Pathophysiology Pediatric Presentation Pharmaceutical Interventions & Vaccines Prevalence and Magnitude Regional Lessons Learned Risk Factors Transmission Patterns Screening & Testing Vulnerable Groups Zoonoses |
| --- | --- |
| Journal websites Searched | Lancet JAMA NEJM Science Nature Eurosurveillance MMWR BMJ CID JID |
| PubMed Query | ("Emerging infectious diseases"[Journal] OR "Lancet (London, England)"[Journal] OR "The Lancet. Infectious diseases"[Journal] OR "The Lancet. Public health"[Journal] OR "The Lancet. Global health"[Journal] OR "The Lancet. Child & adolescent health"[Journal] OR "The Lancet. Psychiatry"[Journal] OR "The Lancet. Haematology"[Journal] OR "The Lancet. HIV"[Journal] OR "The lancet. Gastroenterology & hepatology"[Journal] OR "The Lancet. Planetary health"[Journal] OR "The Lancet. Digital health"[Journal] OR "The Lancet Rheumatology"[Journal] OR "The Lancet. Oncology"[Journal] OR "The Lancet. Neurology"[Journal] OR "The Lancet. Respiratory medicine"[Journal] OR "The lancet. Diabetes & endocrinology"[Journal] OR "JAMA"[Journal] OR "JAMA internal medicine"[Journal] OR "JAMA pediatrics"[Journal] OR "JAMA surgery"[Journal] OR "JAMA cardiology"[Journal] OR "JAMA network open"[Journal] OR "JAMA oncology"[Journal] OR "JAMA neurology"[Journal] OR "JAMA otolaryngology-- head & neck surgery"[Journal] OR "JAMA psychiatry"[Journal] OR "The New England journal of medicine"[Journal] OR "Science (New York, N.Y.)"[Journal] OR "Nature"[Journal] OR "Nature medicine"[Journal] OR "MMWR. Morbidity and mortality weekly report"[Journal] OR "MMWR. Recommendations and reports : Morbidity and mortality weekly report. Recommendations and reports"[Journal] OR "MMWR. CDC surveillance summaries : Morbidity and mortality weekly report. CDC surveillance summaries"[Journal] OR "BMJ (Clinical research ed.)"[Journal] OR "BMJ case reports"[Journal] OR "BMJ open"[Journal] OR "BMJ global health"[Journal] OR "BMJ clinical evidence"[Journal] OR "BMJ quality & safety"[Journal] OR "BMJ innovations"[Journal] OR "BMJ outcomes"[Journal] OR "BMJ open gastroenterology"[Journal] OR "BMJ open sport & exercise medicine"[Journal] OR "BMJ open quality"[Journal] OR "BMJ paediatrics open"[Journal] OR "BMJ sexual & reproductive health"[Journal] OR "BMJ evidence-based medicine"[Journal] OR "BMJ health & care informatics"[Journal] OR "BMJ military health"[Journal] OR "BMJ surgery, interventions, & health technologies"[Journal] OR "BMJ open diabetes research & care"[Journal] OR "BMJ open respiratory research"[Journal] OR "BMJ quality improvement reports"[Journal] OR "BMJ supportive & palliative care"[Journal] OR "Clinical infectious diseases : an official publication of the Infectious Diseases Society of America"[Journal] OR "The Journal of infectious diseases"[Journal] OR "Euro Surveill"[Journal] OR "The Cochrane database of systematic reviews"[Journal]) AND ("coronavirus"[All Fields] OR "ncov"[All Fields] OR "cov"[All Fields] OR "2019-nCoV"[All Fields] OR "COVID-19"[All Fields] OR "SARS-CoV-2"[All Fields]) AND "Journal Article"[Publication Type] |
